# Hormonal Differences in Intimate Partner Violence Perpetrators When They Cope with Acute Stress: A Pilot Study

**DOI:** 10.3390/ijerph18115831

**Published:** 2021-05-28

**Authors:** Ángel Romero-Martínez, Mari-Carmen Blanco-Gandía, Marta Rodriguez-Arias, Marisol Lila, Luis Moya-Albiol

**Affiliations:** 1 Department of Psychobiology, University of Valencia, 46010 València, Spain; marta.rodriguez@uv.es (M.R.-A.); luis.moya@uv.es (L.M.-A.); 2 Department of Psychology and Sociology, University of Zaragoza, 50009 Zaragoza, Spain; mcblancogandia@unizar.es; 3 Department of Social Psychology, University of Valencia, 46010 València, Spain; marisol.lila@uv.es

**Keywords:** acute stress, cortisol, intimate partner violence, oxytocin, testosterone

## Abstract

Background: Only a few studies have paid attention to the ability of perpetrators of intimate partner violence (IPVAW) against women to cope with acute stress, including hormonal parameters. In fact, previous studies assessed how salivary testosterone (Tsal) and cortisol (Csal) changed after coping with an acute emotional stressor (directly related to IPVAW), and they concluded that an imbalance between the two hormones might be characteristic of these men. Nevertheless, they neglected to examine the role of other hormones, such as salivary oxytocin (OXsal), which also seemed to play an important role in behavioral regulation, and whether this response could be generalized to other types of stress not directly related to IPVAW. Methods: This study aims to assess whether IPVAW perpetrators (*n* = 19) present differential hormonal (Tsal, Csal, OXsal and their ratios) and psychological state (anxiety, anger, and general affect) responses when coping with an acute cognitive laboratory stressor (a set of neuropsychological tests performed in front of an expert committee) in comparison with non-violent men (*n* = 16). This quasi-experimental study also assessed whether the psychological state variables drive this different hormonal response. Results: Our results revealed that IPVAW perpetrators had lower Csal and higher Tsal/Csal ratio levels during the post-task period, as well as higher total levels (average) of OXsal than controls. We also found that, only in IPVAW perpetrators, high levels of baseline anxiety and negative affect were related to high rises in Csal during the stress task. Conclusions: These data present a background showing that IPVAW perpetrators and non-violent men cope differently with stress. These findings might help to identify idiosyncratic profiles of IPVAW perpetrators that can then be employed to establish their therapeutic needs. Moreover, we reinforced the importance of combining biological markers with self-reports, thus increasing the reliability of these forensic assessments.

## 1. Introduction

In recent years, numerous researchers have highlighted the importance of including biological markers to obtain a broader comprehension of violence and categorize different profiles of violent individuals in order to design better interventions to reduce violence proneness [1]. However, it is necessary to be cautious about their interpretation and consider other macro-level factors along with these individual-level factors, including biological markers [2,3]. Thus, criminologists currently employ neuroimaging to understand brain correlates of violence proneness. Nevertheless, there are other biological markers that are easier to collect than neuroimaging techniques and also offer valuable information about violence [4]. For example, hormonal parameters, assessed in blood and/or saliva, are relatively easy to collect and analyze. These chemical messengers are released by hormonal glands influencing the nervous system to regulate body physiology and, consequently, influencing human behavior [5,6].

Among violent individuals, there is a growing interest in studying biological correlates that might explain the perpetration of intimate partner violence against women (IPVAW), as well as the consequences for victims’ health [7]. Obviously, these biological correlates should be considered along with other variables, such as psychological, social, and environmental variables to explain IPVAW, which have been proposed as important moderators of IPV perpetration [2,3].

In fact, research assessing IPVAW perpetrators could be divided into two relatively distinct lines. Most studies have been dedicated to employing basal hormonal levels, specifically salivary testosterone (Tsal) and cortisol (Csal), as a ‘trait’ related to violence proneness in IPVAW perpetrators [8,9,10,11,12], with higher Tsal levels normally being related to low marital quality and high violence. Moreover, high Tsal and low Csal levels, as well as a high Tsal/Csal ratio, have been associated with personality traits such as borderline, antisocial, or narcissistic personalities, which subsequently explained the risk of being involved in antisocial situations such as drug misuse or IPVAW recidivism [12,13,14]. Unfortunately, these studies had an important methodological limitation. That is, researchers only collected a single sample during a day, which entailed low reliability of the hormone measurements. This is explained, at least in part, by the fact that Tsal and Csal levels tend to fluctuate across a day (circadian pattern) and seasonally. Furthermore, these hormones are affected by daily stressors [6]. Therefore, the association between hormones and personality traits, although significant, would be spurious and/or relatively questionable.

The other line of research has tried to solve the low reliability of hormonal measurements by testing dynamic endocrine functioning, continuously collecting saliva samples during laboratory stress tasks. Two studies assessed whether IPVAW perpetrators’ Tsal and Csal responses to an emotional stressor related to IPVAW (talking about their criminal records and their opinion of the IPVAW law) along with another purely cognitive task (an arithmetic task) differ from non-violent men’s responses to this task. These studies found significant hormonal differences between groups at specific moments [15,16,17]. Even though the Tsal response to the stress task was similar for both groups, IPVAW perpetrators presented higher levels of Tsal during the preparatory period and immediately after the stress task. Moreover, the Csal levels of the IPVAW perpetrators did not vary across the stress task, unlike controls, who experienced variations in their Csal levels [16,17]. They also found group differences after calculating the quotient between the Tsal and Csal levels. In this regard, it seems that the higher the differences between the levels of the previously mentioned hormones (imbalance), the higher the anger proneness would be [15]. Nevertheless, the imbalance between these hormones does not directly impact behavior. In fact, it could interfere in violence proneness by affecting emotional processing. For example, it seems that the increase in endogenous testosterone (T) levels might reduce the accuracy in general emotion processing, whereas cortisol (C) and Oxytocin (OX) tend to enhance emotional processing accuracy. Moreover, it has been previously established that increases in T enhance accuracy for angry faces, but OX diminishes accuracy for this emotion [18].

Recently, it was suggested that a neuropeptide known as oxytocin plays an important role in facilitating prosocial behaviors such as love, bonding parenting, and violence, among others [19]. Furthermore, this hormone seems to interact with T and C [18]. Thus, even though animal and human studies initially concluded that higher levels of these hormones are related to prosocial behaviors, a large number of manuscripts have reported exactly the opposite result, or even an absence of significant results beyond prosociality [19,20]. In any case, the interactions between these hormones might modulate approaching or avoiding violence, obviously interacting with or being modulated by other non-hormonal variables [18,19,20,21]. Hence, it would be useful to assess how these hormonal variables interact by calculating statistical associations and/or the quotients between hormonal levels [22].

Regarding IPVAW perpetrators, only two studies have assessed how oxytocin is related to IPVAW perpetration in laboratory contexts. The first study concluded that administration of OX only increased IPVAW proneness and arguments with their partners in highly physically violent individuals [23]. However, a later study that employed the same procedure failed to report variations in IPVAW proneness in men after OX administration [24]. Moreover, this study incorporated the Csal response into this laboratory task to assess whether it interacts with OX variations. However, they did not find any significant interactions between these hormones in men. Unfortunately, these studies neglected to include a non-violent control group or, more importantly, the interaction between salivary OX (OXsal) and Tsal and Csal in laboratory sessions unrelated to IPVAW.

Because previous literature in this field employed laboratory tasks related to IPVAW, which might be an emotionally biased task for IPVAW perpetrators [14,15,16], we decided to employ a stressor that affects all participants equally (performing a set of neuropsychological tests in front of a committee of experts who provide feedback about their performance), thus avoiding any emotionally biased topics for both groups. This acute laboratory stress task has demonstrated its usefulness in promoting salivary hormonal and psychophysiological variations in several groups of non-violent and violent individuals, including IPVAW perpetrators [25,26,27,28,29].

To address this gap in the literature, this study aimed to explore whether Tsal, Csal and Oxsal responses of IPVAW perpetrators, as well as their ratios (quotients between different hormonal levels), to an acute laboratory stressor differ from those of non-violent men. Based on previous scientific evidence with IPVAW perpetrators in response to acute stress [14,15,16,21,22,27], as well as interactions between the hormones described above [15,16,17], we hypothesized that IPVAW perpetrators would present higher Tsal and Tsal/Csal ratios as well as lower Csal and OXsal than controls in response to an acute validated stressor. Additionally, this study investigated the potential relationship between the previously mentioned hormones and affective states in response to acute stress. Based on previous results, heightened negative affect is related to high Tsal levels [14,15]. For this reason, we expected that, in both groups, negative affect would be positively related to baseline Tsal and negatively to Csal and OXsal.

## 2. Materials and Methods

### 2.1. Participants

From an initial sample of 50 healthy men who initially agreed to participate in the study, only 40 participants were included in the statistical analysis because 10 refused to provide biological samples. In fact, we removed five participants (one IPVAW perpetrator and four controls) because they were outliers (>2.5 SD from group mean) for Csal and Tsal (19 IPVAW perpetrators and 16 controls) (see Table 1). The IPVAW volunteers come from the CONTEXTO psycho-educational and community-based treatment program at the University of Valencia. This intervention was designed for men convicted of IPVAW. These men received a suspension of their sentence on the condition that they attend this intervention. Nevertheless, to receive this suspension, it is necessary to have a sentence of less than two years and no previous criminal record directly related to IPVAW or other kinds of criminal acts [30]. Volunteers were screened to include only those participants without physical (e.g., strokes, chronic pain, traumatic brain injuries…) or mental disorders or drug misuse (lower scores than cutoff scores on the Alcohol Use Disorders Identification Test for alcohol and the Severity Dependence Scale for cannabis and cocaine [31,32,33]. Moreover, we also screened for personality disorders by applying the MILLON-III. In fact, all the included participants scored below 35 on antisocial, narcissistic, and/or borderline disorders [34].

Regarding controls, we posted advertisements for male volunteers in the province of Valencia. We provided a telephone number and an email address to contact us. After expressing interest in participating in our study, we screened for participants with similar anthropometric and demographic characteristics to those of the IPVAW perpetrators, that is, an absence of physical or mental disorders and drug misuse. Moreover, it was also necessary to present an official certificate showing the absence of a criminal record. Lastly, we also included those volunteers with a score below 1 on psychological abuse and physical assault on conflict tactics [35,36].

After agreeing to participate in our study, volunteers signed an informed consent, which was elaborated according to the Helsinki Declaration and approved by the University of Valencia Ethics Committee (code: H1348835571691).

### 2.2. Procedure

Participants were initially screened in a telephone interview to analyze their suitability for the study. Those who presented the appropriate profile completed a session in the psychobiology laboratories of the University of Valencia after signing the informed consent. This session took place between 4:00 and 7:00 p.m., based on recommendations in previous literature to assess hormonal levels in the afternoon because Csal levels are relatively stable [26]. Once participants had arrived at the laboratory, they were taken to a noise-insulated room with a constant temperature of 22 ± 1 °C. During the entire experimental session, which lasted approximately 90 min, participants remained seated, and saliva samples were collected at specific ‘times’ (e.g., resting, preparatory, post-task, and + 20 min post-task). Furthermore, their psychological state (specifically, positive and negative affect) was assessed before and after the stress task.

To avoid potential biases in the topic employed as a stressor and based on previous research in this field [27,28,37,38,39], we decided to include a stress task consisting of a battery of neuropsychological tests in the following order: memory, attention, and executive functioning. Participants completed these tests in front of an audience consisting of two evaluators (a man and a woman). We provoked a socio-evaluative threat by offering feedback about their performance (e.g., “Could you try harder to do the tasks?”, “Can you just do that?”, “Is that all you can do?”, “Your colleagues scored higher on these tasks”, “We recommend that you try harder”, etc.). By offering constant, negative feedback during their performance, we promoted high socio-evaluative stress. As previously recommended [37,38,39], we provided this feedback at specific moments, equally for all the individuals submitted to this stressor. Once participants had finished the stress task, they were asked several questions related to this task. Initially, they were asked about the level of perceived stress, rated from 0 (absence) to 10 (extremely stressful). In the same way, they were asked about their level of satisfaction with their performance on the stress task, rated from 0 (dissatisfied) to 10 (highly satisfied). Lastly, we assessed whether participants thought their performance was explained by internal (e.g., personal effort and mental abilities to solve neuropsychological tests) attribution of the outcome during the stress task, and scores ranged from 0 (low internal locus of control) to 10 (high internal locus of control).

### 2.3. Psychological State Variables

We employed the translated and validated version of the “State-Trait Anxiety Inventory” (STAI-S) to assess state anxiety [40,41]. This test consists of a set of 20 items rated on a 4-point Likert scale. The reliability coefficients for this study were 0.65 and 0.73 for baseline and post-task measures, respectively.

State anger was measured by the State-Trait Anger Expression Inventory-2’ (STAXI-2) [42], translated and validated in Spanish [43]. It consists of 15 items rated on a 4-point Likert-type scale (1 = ‘not at all’ to 4 = ‘very much so’). A total score was obtained by adding up the scores on each item. Cronbach’s alphas were 0.99 and 0.88 for baseline and post-task assessments, respectively.

We employed the PANAS Scales of Positive and Negative Affect [44] as a measure of affectivity. This questionnaire consists of 20 items, half of them assessing positive affect and the other half negative affect. All of the items were rated on a 5-point Likert-type scale (1 = ‘not at all’ to 5 = ‘very much so’). Total positive and negative affect scores were calculated before and after the laboratory task. Cronbach’s alphas were 0.77 and 0.56 for positive affect baseline and post-task measures, respectively. Moreover, reliability was 0.63 and 0.73 for negative affect baseline and post-task measures, respectively.

### 2.4. Hormone Measurements

We collected saliva directly from the mouth using Salivette devices for Csal and OXsal (Sarstedt, Rommelsdorf, Germany) and sterile glass tubes for Tsal samples. Participants were advised about the importance of avoiding brushing their teeth and eating or drinking stimulants two hours before the laboratory procedure. After collecting saliva samples always in the same order (first those for assessing Csal and OXsal, and then those for Tsal), the samples were frozen at −20 °C until posterior analysis.

Csal and Tsal levels were assessed by the 96-well ELISA Kit (ab154996 for Csal and ab178655 for Tsal, Abcam, Cambridge, UK), whereas for OXsal the Oxytocin EIA kit (Arbor Assays, Inc. Ann Arbor, MI, USA; ref: K048) was employed. Regarding assessment sensitivity, for the Csal ELISA Kit, it was 0.12 ng/mL, and for Tsal, it was 2.96 pg/mL. To measure Oxsal, saliva samples were initially lyophilized (Modulyo Freeze Dryers, Thermo Electron Corporation) for approximately 15 h and, afterwards, dehydrated. Then, they were reconstituted in 250 μL of assay buffer, which produced a concentration four times higher than the original. This allowed them to fall within the kit’s sensitivity range and be detectable on the standard curve. Neuropeptide cross-reactivity was reported by Arbor assays as <0.001%, and the detection limit was 11 pg/mL. Inter-trial and inter-trial CV averaged less than 10%.

All hormonal levels were expressed in the same units (pg/mL), which allows us to calculate the ratio between them, that is, the quotients between Tsal, Csal, and OXsal (Tsal/Csal, Tsal/OXsal, and Csal/OXsal ratios).

### 2.5. Data Analysis

We employed Shapiro-Wilk tests to check whether the variables were normally distributed. Because most of the hormonal variables did not meet the assumption of normality (significance equal to or below 0.05), we decided to log-transform these variables. However, for the anthropometric (e.g., age and body mass index) and sociodemographic variables and appraisal assessment, t-tests were conducted with Levene’s test for equality of variances and/or Chi square analyses to assess group differences. Cohen’s d was calculated to provide effect sizes for the between-group differences [45].

To explore hormonal and psychological state changes in response to the laboratory task, we performed repeated measures ANOVA with ‘time’ (baseline, preparatory, stress, and recovery) for hormones and ‘time’ (pre and post) for psychological variables as the within-subject factor and ‘group’ (IPVAW and controls) as the between-subject factor for the whole sample. Then, t-tests were performed as post-hoc tests for variables that yielded significant results. Greenhouse-Geisser corrections were performed for degrees of freedom. For significant results, partial eta squared (ηp^2^) is reported as a measure of effect size. Furthermore, we also calculated the statistical power for significant post-hoc results.

The magnitudes of the stress responses for the hormones included in this study were estimated by the area under the curve with respect to the increase (AUCi) and to the ground (AUCg), based on the trapezoidal formulae [46]. Whereas the AUCi is calculated with respect to the baseline measurement, the AUCg is considered the distance from zero.

For psychological state variables, we calculated the change score of each scale by using resting to post-task to baseline scores. T-tests were conducted with Levene’s test for equality to test for potential group differences.

Correlational analysis was performed to assess whether there were associations between the variables (psychological state baseline and change score with hormonal baseline levels and AUC of each hormone). Nevertheless, we think it would be appropriate to run Bonferroni corrections, considering only relationships with a *p* value lower than or equal to 0.001 significant.

Finally, data analyses were performed using SPSS 26.0 (SPSS IBM). All reported *p*-values were two-tailed, and *p* ≤ 0.05 was considered significant. Average values are expressed as mean ± SD and mean ± SEM.

## 3. Results

### 3.1. Participant Characteristics and Appraisal Scores

When assessing group differences in anthropometric and demographic variables, no group differences were found (see Table 1).

Regarding appraisal assessment, IPVAW perpetrators did not differ from controls on perceived stress (t(33) = 0.693, *p* = 0.697; 2.63 ± 2.27 and, 2.97 ± 2.81, respectively), satisfaction (t(33) = −0.488, *p* = 0.628; 6.45 ± 2.26 and, 6.15 ± 0.81, respectively), or the internal locus of control (t(33) = 0.809, *p* = 0.424; 5.65 ± 3.05 and, 6.37 ± 1.95, respectively).

### 3.2. Group Differences in Response to the Laboratory Task (Hormonal and Psychological State Variables) 

**Figure 1:**
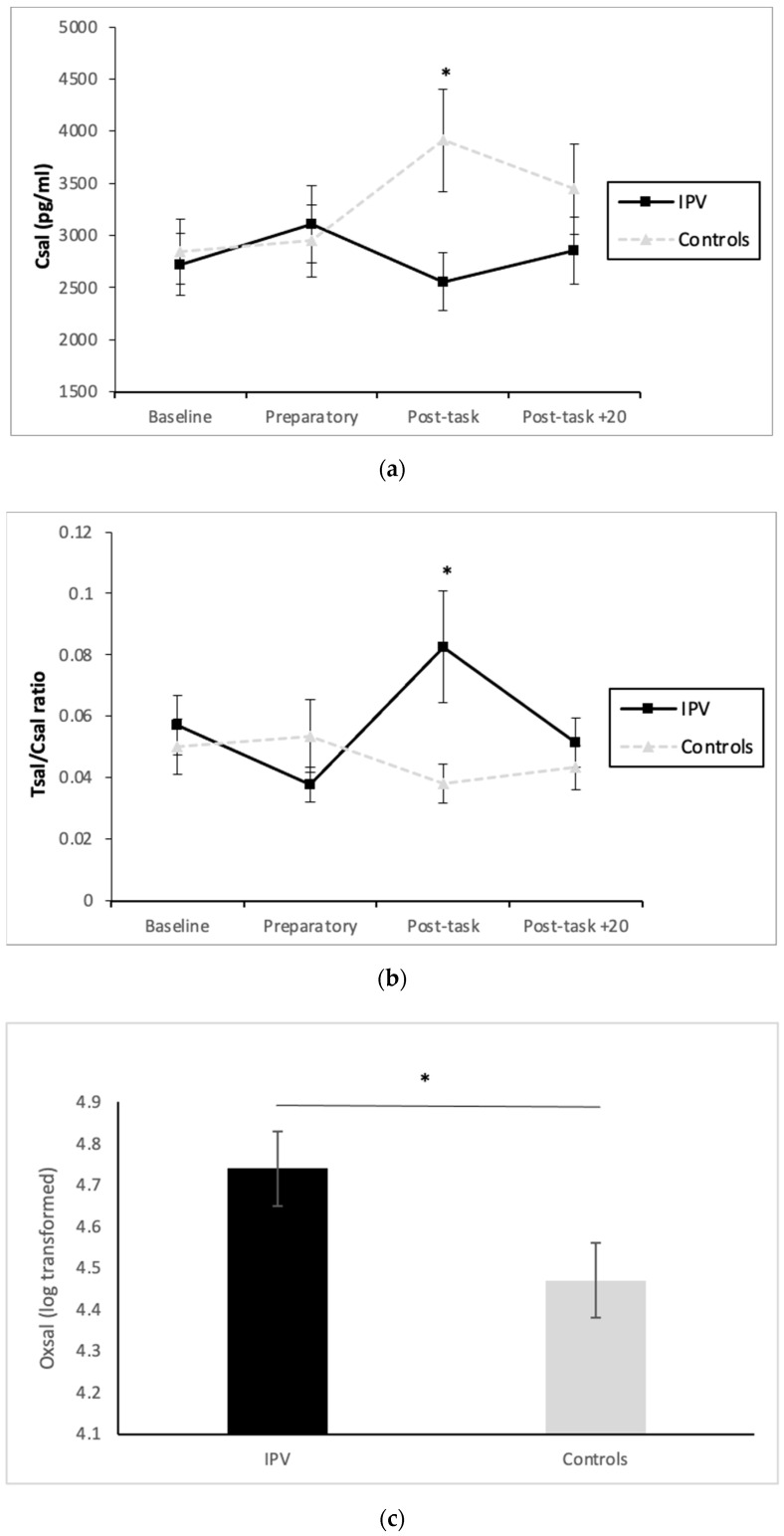
Csal (**a**), T/C ratio (**b**), and (**c**) Oxsal levels for IPVAW and control groups. Note. Csal: Salivary Cortisol; Csal/Tsal ratio; Salivary Cortisol and Testosterone ratio; IPVAW: Intimate Partner Violence; OXsal: Salivary Oxytocin. * *p* < 0.05.

Initially, no significant ‘time’ effects were found for any hormonal variable included and/or their ratios (*p* > 0.05). Nonetheless, significant ‘time x group’ interactions were found for Csal [F(3, 99) = 2.71 *p* = 0.049; η2 = 0.076, Figure 1a) and the Tsal/Csal ratio [ε = 0.57, F(1.70, 56.20) = 3.89 *p* = 0.032; η2 = 0.105, Table 2 and Figure 1b]. Post-hoc t-tests confirmed that groups differed on their Csal and Tsal/Csal ratio levels, specifically at post-task (t(23.86) = −2.39, *p* = 0.025, d = 0.83, 1352.73 ± 563.79, 95% CI = −2516 to −188) and t(22.29) = 2.29, *p* = 0.031, d = 0.64, −0.044 ± 0.019, 95% CI = 0.004 to 0.08). Specifically, IPVAW perpetrators presented lower Csal and higher Tsal/Csal ratio levels at post-task. Moreover, we also found a significant ‘group’ effect for OXsal [F(1, 33) = 4.47, *p* = 0.042, η2 = 0.12], with IPVAW perpetrators presenting higher total OXsal levels than controls (4.74 ± 0.09 and 4.47 ± 0.09, respectively) (Table 2 and Figure 1c). Statistical power for each post-hoc test was 1.

Regarding STAI-S, a significant ‘time’ effect was only found for STAI-S [F(1, 33) =  7.24, *p*  = 0.011; η2 = 0.180), with IPVAW perpetrators and controls presenting higher levels before the laboratory task (24.73 ± 4.70 and 23.94 ± 5.17, respectively) than after it (21.44 ± 5.57 and 22.94 ± 5.16, respectively). However, no significant ‘time’ or ‘time x group’ effects were found for STAXI-2 S or PANAS (positive or negative affect). Additionally, we did not find differences between groups in change scores for STAI-S (t(33) = 0.934, *p* = 0.357), STAXI-2 (t(33) = −1.75, *p* = 0.089), PANAS positive (t(33) = −1.33, *p* = 0.193), or PANAS negative affect (t(33) = 0.208, *p* = 0.837) (Table 3).

No significant relationships were found between variables for the whole sample. Therefore, we decided to divide the sample into two groups. After splitting it into two groups, we assessed the relationship between the above-mentioned variables, considering only those correlations with a *p* value lower than or equal to 0.001 significant. In this regard, we only found significant correlations for IPVAW perpetrators. Specifically, a significant association was found between Csal baseline levels and STAI-S baseline (r = 0.686, *p* = 0.001). Moreover, we also found a significant association between the PANAS negative affect baseline and Csal AUCi (r = 0.646, *p* = 0.001), as well as between the Csal/OXsal ratio baseline and STAXI-2 baseline (r = 0.868, *p* < 0.001) and the STAXI-2 change score (r = −0.849, *p* < 0.001) (Table 4).

## 4. Discussion

This is one of the first studies to investigate changes in specific hormonal parameters (including Tsal, Csal, and OXsal as well as their ratios) in response to acute stress (without emotional biases) in a group of IPVAW perpetrators in comparison with controls. Our data revealed that IPVAW perpetrators had lower Csal and higher Tsal/Csal ratio levels than controls in response to acute stress immediately after the stress task, that is, during the post-task period. Moreover, IPVAW perpetrators presented higher total levels (average) of OXsal than controls. It is necessary to highlight that the statistical power for the post-hoc test was adequate. After splitting the sample into groups to assess the relationships between the variables, in IPVAW perpetrators, higher Csal baseline levels were related to higher baseline anxiety levels. Furthermore, the higher the negative baseline affect, the higher the Csal increases during the laboratory stress task. Finally, we also found that higher Csal/OXsal ratio baseline levels were related to higher state anger baseline levels and lower change during a laboratory task of state anger.

The stress protocol included in this study has shown its suitability for promoting hormonal changes in previous studies with non-violent individuals [25,27,28,29], even cardiorespiratory and electrodermal variations in IPVAW perpetrators [47]. Nonetheless, the current study did not support this because a significant ‘time’ effect was not obtained for the hormonal variables. The absence of a significant ‘time’ effect in this study might be explained by the relatively low number of saliva samples collected. For example, we only collected four saliva samples, whereas previous studies collected more than eight samples during laboratory stress tasks [25,27,28,29]. Moreover, psychophysiological variables are more sensitive to time changes because they are continuously registered during a sustained period (Romero-Martínez et al., in press). Most importantly, the absence of a ‘time’ effect could not be explained by the stress perception (appraisal assessment) because the groups did not differ. Furthermore, the current assessment of appraisal (stress perception and satisfaction) was similar to previous studies that obtained significant ‘time’ effects employing the same stress protocol [25,47].

The fact that the groups only differed in a specific time, such as post-task on the Csal and Tsal ratio, partly supported previously published research in this field [15,16,17]. We previously hypothesized that IPVAW perpetrators would present a higher Tsal/Csal ratio because these individuals present higher imbalances between the two hormones. In fact, the current study also demonstrated a higher ratio, but it also differed in Csal levels instead of Tsal levels, as previously pointed out [15,16]. Differences between previous research and the current study would be explained by the laboratory stress task employed; that is, a psychosocial stressor based on an emotionally biased topic was previously employed. More importantly, this study extended previous results, concluding that there was a different physiological pattern of response to coping with stress in general that was not explained by an emotional stress topic, differential appraisal assessments, or the presence of drug misuse.

The main novelty of this research is the assessment of OXsal and how this hormone varies in response to acute stress. It has been hypothesized that violent individuals are characterized by flattened or even hypoactive functioning of the hypothalamus-pituitary-adrenal axis, which is unable to control T production through its inhibiting effect on the hypothalamus. This would explain the higher levels of T (imbalance between these hormones) observed in violent [48] and chronically stressed individuals, with hormonal imbalance also being associated with anger proneness [15,16,17]. OXsal tends to be positively related to Tsal and inversely to Csal [18,19]. Hence, our data revealed that IPVAW perpetrators presented higher total OXsal levels than controls, which might be explained by the low levels of Csal in this group. Unfortunately, correlational analysis did not support significant relationships between the hormones. Despite the absence of significant relationships between the hormonal variables, it might be possible to explore other biochemical ways the three hormones and subsequent violence proneness are related. Moreover, it is particularly important to highlight that we are working with the free fraction of hormones in saliva, which tends to differ from central nervous system accounts or those parts that play an active role. In any case, our data are congruent with a previous study, and they establish that increased OX levels were related to high IPVAW, particularly in highly aggressive individuals [23]. However, it is particularly important to mention that these hormones tend to indirectly affect aggressive behavior by modulating, for example, emotional processing [2]. Hence, further research should explore in what ways hormones explain violence proneness, in order to design better interventions by interfering in these systems. In fact, it would be interesting to include other hormonal parameters such as vasopressin, which closely interacts with OX. Lastly, we would like to highlight that it would be a mistake to establish reductionist explanations for the association between these hormones and violence proneness, with endogenous levels interacting at different levels in approaching and avoiding different behaviors.

Curiously, we did not find differences in the psychological state response to the laboratory stress task (e.g., anxiety, anger, or negative affect), even though state anxiety levels and negative affect were only related to Csal changes in response to the laboratory stress task in IPVAW perpetrators. This might explain, at least in part, specific Csal differences between groups in response to an acute laboratory stressor. This is partly consistent with previous results in this field. Only in IPVAW perpetrators were hormonal variables related to psychological state variables [16].

The current study makes a considerable contribution to this field of research, but several limitations should be taken into account in interpreting our data. The first one is the small sample size; thus, our findings should be considered a pilot study to guide future research. Nevertheless, this limited sample size could be compensated by the large number of saliva samples collected or the use of a non-emotionally biased laboratory stress task. Furthermore, the assessment of endogenous levels of OXsal might offer a broader understanding of violence proneness. In any case, additional research is needed to explore these patterns in larger samples. Second, the characteristics of the participants included in this study limited the external validity of our results, making it necessary to include a more heterogeneous sample with different profiles in future research. Third, the assessment of a single session was an important limitation to the reliability of the biological measurements. To understand whether differences between participants could be explained by a differential allostatic load, it would be particularly important to collect saliva samples in different sessions, including five weekdays to increase the reliability of the hormonal measurements. Fourth, the correlational and uncontrolled nature of this study limited the establishment of causal associations between variables. Therefore, it is important to be cautious about the interpretation of our data. However, our data are novel because only a few studies have paid attention to these biological parameters and their interrelationships. This study is part of an ongoing research effort to improve our understanding of how IPVAW perpetrators cope with acute stress. Additionally, we also consider it particularly important to find out whether these biological parameters allow us to differentiate among different types of IPVAW perpetrators (e.g., family only vs those who perpetrate violence against others; psychopathic profiles…), or even compared to other subsamples of violent individuals of both genders. Finally, it is particularly interesting to highlight that future studies should attempt to replicate these results by comparing different types of laboratory tasks, some related to IPVAW and others unrelated. This would make it possible to standardize laboratory procedures and reinforce their reliability for future research.

## 5. Conclusions

In summary, this manuscript reveals the existence of hormonal differences between IPVAW perpetrators and controls in coping with stress, although it is necessary to keep in mind that these differences are subtle and temper the generalization of our conclusions. This research represents an advance in the comprehension of how these men cope with acute stress, and it shows that their profile is somewhat different from that of non-violent men. Our results provide evidence to support the hypothesis of the existence of an imbalance between Tsal and Csal in violent populations, with these individuals also differing in other pituitary hormones such as OX. This reinforces the need to include several biological parameters and their associations to understand violence proneness, instead of isolated biological markers. Thus, it would be necessary to include hormonal parameters to develop and understand IPVAW perpetrators’ ability to cope with acute stress. These correlates of behavioral regulation might be useful for studying potential changes in IPVAW perpetrators after intervention programs. These biological markers also could be employed as complements to profiling in IPVAW perpetrators. For example, hormonal markers might be combined with other personality and/or psychological variables to build IPVAW typologies to predict treatment adherence and risk of IPVAW recidivism.

## Figures and Tables

**Table 1:** Means (Standard Deviations), Percentages, and Means Comparisons for Anthropometric (age and body mass index) and Demographic Variables in All Groups.

	IPVAW Perpetrators (*n* = 19)	Controls (*n* = 16)	*t*-Test/Chi-Square	Significance (*p* Value)
Age (*M, SD*)	39.68 (10.24)	41.43 (8.39)	−0.55	0.588
Body mass index	24.66 (3.73)	27.25 (6.86)	−1.41	0.165
Marital status (%)				
Married	11	25	1.28	0.351
Single, divorced or separated	89	75		
Level of education (%)				
Primary/lower secondary	79	56	3.38	0.185
Upper secondary/vocational training	21	31		
University	-	13		
Employment status (%)				
Employed	84	63	2.14	0.245
Unemployed	16	37		
Number of offspring (M, SD)	1.21 (1.22)	0.57 (0.63)	1.91	0.065

**Table 2:** Means and Standard Error of the Mean for Hormones in Each Group.

	IPVAW Perpetrators (*n* = 19)	Controls (*n* = 16)	Mean Difference	CI (95%)	F ANOVA (Time × Group)	Significance (*p* Value)	Partial Eta Squared (η_p_^2^)
Tsal (pg/mL)							
Baseline	141.58 (22.04)	131.81 (20.51)	9.77	−52 to 71	0.58	0.60	0.02
Preparatory	107.58 (0.007)	127.81 (19.67)	−20.23	−0.75 to 34			
Post-task	137.22 (16.74)	126.50 (17.85)	10.72	−39 to 60			
Post-task +20	122.57 (15.90)	131.37 (22.23)	−8.79	−64 to 47			
Csal (pg/mL)							
Baseline	2.722 (297.12)	2.848 (311.93)	−126.64	−1005 to 752	2.71	0.05	0.08
Preparatory	3.108 (372.22)	2.947 (345.55)	161.18	−886 to 1209			
Post-task	2.557 (274.58)	3.910 (492.50) *	−1352.73	−2516 to −188.75			
Post-task +20	2.855 (325.36)	3.446 (433.33)	−591.87	−1700 to 516			
OXsal (log-transformed)							
Baseline	4.67 (0.100)	4.62 (0.162)	0.05	−0.32 to 0.42	0.99	0.40	0.03
Preparatory	4.86 (0.096)	4.51 (0.184)	0.35	−0.05 to 0.76			
Post-task	4.73 (0.132)	4.22 (0.179)	0.50	0.04 to 0.96			
Post-task +20	4.72 (0.115)	4.55 (0.238)	0.17	−0.38 to 0.72			
Tsal/Csal ratio							
Baseline	0.06 (0.009)	0.05 (0.009)	0.01	−0.02 to 0.03	3.89	0.03	0.11
Preparatory	0.04 (0.005)	0.05 (0.011)	−0.02	−0.04 to 0.009			
Post-task	0.08 (0.018)	0.04 (0.006) *	0.04	0.004 to 0.08			
Post-task +20	0.05 (0.008)	0.04 (0.007)	0.01	−0.01 to 0.03			
Tsal/OXsal ratio							
Baseline	1.57 (0.325)	1.52 (0.321)	0.05	−0.88 to 0.99	1.01	0.36	0.03
Preparatory	0.95 (0.213)	2.52 (1.249)	−1.56	−3.93 to 0.80			
Post-task	1.31 (0.222)	2.12 (0.629)	−0.81	−2.10 to 0.49			
Post-task +20	1.19 (0.206)	2.03 (0.755)	−0.84	−2.49 to 0.81			
Csal/OXsal ratio							
Baseline	29.98 (6.618)	34.85 (6.647)	−4.87	−24 to 14	0.67	0.47	0.02
Preparatory	26.21 (4.254)	58.28 (29.87)	−32	−88 to 24			
Post-task	28.39 (6.713)	56.04 (11.140)	−27	−53 to −2.10			
Post-task +20	30.39 (6.614)	46.71 (12.488)	−16	−43 to 10			

Note. Csal: Salivary Cortisol; IPVAW: Intimate Personal Violence; OXsal: Salivary Oxytocin; Tsal: Salivary Testosterone. * *p* < 0.05.

**Table 3:** Means and Standard Error of the Mean for Hormones and Psychological state variables in Each Group.

	IPVAW Perpetrators (*n* = 19)	Controls (*n* = 16)	Mean Difference	CI (95%)	F ANOVA (Time × Group)	Significance (*p* Value)	Partial Eta Squared (η_p_^2^)
STAI-S							
Baseline	24.74 (1.08)	23.94 (1.29)	0.79	−2.59 to 4.19	3.08	0.09	0.09
Post-task	24.21 (1.04)	21.44 (1.39)	2.77	−0.70 to 6.25			
STAXI-2							
Baseline	17.79 (2.38)	15.31 (0.17)	2.47	−2.82 to 7.78	0.87	0.36	0.03
Post-task	15.31 (0.18)	15.31 (0.19)	0.003	−0.62 to 0.63			
PANAS							
Negative affect							
Baseline	12.68 (0.83)	13.06 (0.71)	−0.37	−2.65 to 1.89	0.04	0.83	0.00
Post-task	12.42 (0.63)	13.06 (1.28)	−0.64	−3.41 to 2.12			
Positive affect							
Baseline	30.84 (1.35)	29.63 (1.54)	1.22	−2.93 to 5.37	1.77	0.19	0.05
Post-task	33.67 (3.09)	27.94 (2.03)	5.43	−2.12 to 12.98			

Note. IPVAW: Intimate Personal Violence against Women.

**Table 4:** Relationships of hormonal variables (baseline and AUC) with psychological state assessment (baseline and change score) for IPVAW perpetrators and controls applying Bonferroni correction.

	STAI-S Baseline		STAI-S Change Score		STAXI-2 Baseline		STAXI-2 Change Score		PANAS Negative Baseline		PANAS Negative Change Score		PANAS Positive Baseline		PANAS Positive Change Score	
	IPVAW Controls		IPVAW Controls		IPVAW Controls		IPVAW Controls		IPVAW Controls		IPVAW Controls		IPVAW Controls		IPVAW Controls	
Tsal baseline	−0.346	−0.592	0.262	0.121	0.275	0.002	−0.236	−0.106	−0.126	−0.307	0.168	0.054	0.162	−0.342	−0.166	0.087
Tsal AUCi	0.086	0.391	0.105	−0.127	−0.260	−0.127	0.255	0.242	0.337	0.110	−0.317	0.065	−0.496	0.110	0.156	0.076
Tsal AUCg	0.157	0.386	0.105	−0.221	−0.209	−0.227	0.211	0.229	−0.335	−0.007	0.384	−0.104	−0.444	−0.158	0.122	0.257
Csal baseline	0.686 **	−0.161	−0.662	0.204	−0.057	−0.102	−0.390	−0.067	−0.108	−0.305	−0.285	0.139	0.150	−0.034	−0.111	−0.028
Csal AUCi	0.082	0.119	0.352	0.265	−0.344	−0.040	0.318	0.080	0.646 **	0.171	−0.332	0.137	−0.438	0.204	0.022	0.039
Csal AUCg	0.136	0.199	0.393	0.219	−0.237	−0.146	0.206	0.224	0.530	0.107	−0.271	0.139	−0.332	0.022	0.083	0.128
OXsal baseline	−0.321	−0.160	−0.175	−0.192	−0.451	−0.603	0.435	0.424	−0.054	0.253	0.237	−0.046	−0.290	−0.076	0.221	−0.172
OXsal AUCi	0.382	0.246	0.195	0.175	0.295	0.145	−0.296	−0.027	0.057	0.135	−0.193	0.109	0.228	0.292	−0.013	0.150
OXsal AUCg	0.326	0.236	0.208	0.099	0.186	0.004	−0.185	−0.027	0.190	0.070	−0.088	0.111	0.118	0.333	−0.165	0.062
Tsal/Csal ratio baseline	−0.117	−0.466	0.418	−0.061	−0.025	0.018	0.031	−0.056	0.138	−0.144	0.243	−0.007	−0.001	−0.276	−0.086	0.172
Tsal/Csal ratio AUCi	−0.186	−0.425	0.413	0.125	0.013	0.147	−0.017	−0.044	0.039	−0.100	0.272	0.095	0.076	−0.152	−0.005	0.387
Tsal/Csal ratio AUCg	−0.264	−0.425	0.054	−0.032	0.213	0.107	−0.173	0.199	−0.250	0.061	0.316	−0.175	−0.036	−0.464	−0.091	0.126
Tsal/OXsal ratio baseline	−0.105	−0.345	0.092	0.140	0.603	0.511	−0.565	−0.448	−0.102	0.031	0.014	0.046	0.278	−0.166	−0.226	0.025
Tsal/OXsal ratio AUCi	−0.546	−0.367	0.400	0.258	−0.052	−0.054	0.118	−0.009	0.209	−0.153	0.010	−0.131	−0.275	−0.071	−0.212	0.112
Tsal/OXsal ratio AUCg	−0.482	−0.274	0.523	0.116	−0.014	−0.001	0.065	−0.132	0.083	−0.119	−0.073	−0.042	−0.357	−0.010	−0.138	0.041
Csal/OXsal ratio baseline	0.172	−0.061	−0.325	0.174	0.868 **	0.420	−0.849 **	−0.418	0.442	0.006	−0.246	0.067	0.253	−0.035	−0.153	0.032
Csal/OXsal ratio AUCi	−0.028	−0.284	0.268	0.260	−0.084	−0.058	0.098	−0.054	−0.085	−0.160	−0.451	−0.084	−0.392	0.024	−0.200	0.093
Csal/OXsal ratio AUCg	−0.127	−0.197	0.147	0.244	0.183	0.135	−0.171	−0.287	0.050	−0.043	−0.162	−0.039	−0.184	0.118	−0.138	0.067

Note. AUC: Area Under the Curve; Csal: Salivary Cortisol; IPVAW: Intimate Personal Violence Against Women; OXsal: Salivary Oxytocin; Tsal: Salivary Testosterone. ** *p* ≤ 0.001.
